# Facilitators and barriers to physical activity in middle-aged and older adult(s) HIV infected persons: a systematic review of qualitative studies

**DOI:** 10.3389/fpubh.2026.1809117

**Published:** 2026-06-02

**Authors:** Yali Zhou, Xutong Zheng, Dan Shao, Fang Cheng

**Affiliations:** 1 Wuhan Jinyintan Hospital, Tongji Medical College of Huazhong University of Science and Technology, Hubei Clinical Research Center for Infectious Diseases, Wuhan, China; 2 Wuhan Research Center for Communicable Disease Diagnosis and Treatment, Chinese Academy of Medical Sciences, Wuhan, China; 3 Joint Laboratory of Infectious Diseases and Health, Wuhan Institute of Virology and Wuhan Jinyintan Hospital, Chinese Academy of Sciences, Wuhan, China; 4 Department of Public Service, The First Hospital of China Medical University, Shenyang, China

**Keywords:** aged, exercise, HIV, physical activity, qualitative research

## Abstract

**Purpose:**

This study aims to objectively evaluate and synthesize qualitative studies on the facilitators and barriers to PA among middle-aged and older adults living with HIV.

**Methods:**

A systematic search was conducted in four databases—Web of Science, Embase, PubMed, and CINAHL—covering the period from January 2000 to December 2024. The included studies were qualitatively reviewed according to the Enhancing Transparency in Reporting the Synthesis of Qualitative Research (ENTREQ) guidelines. A meta-aggregative approach was used to summarize and categorize the identified facilitators and barriers.

**Results:**

A total of 9 articles were included in the study, identifying 71 facilitators and 59 barriers. These factors were categorized into two main themes and seven sub-themes.

**Conclusion:**

PA among middle-aged and older adults living with HIV is influenced by multiple factors. There is a need to deeply understand the actual situation of this population and focus on the core elements affecting PA in order to improve the health of middle-aged and older adults living with HIV and help address the global issue of HIV and aging.

**Systematic review registration:**

Identifier: CRD42024498010.

## 1 Introduction

Recent studies indicate that individuals living with HIV often engage in lower levels of physical activity (PA), which can lead to further health complications (1). According to data from the World Health Organization (WHO), by the end of 2022, approximately 39 million people worldwide were living with human immunodeficiency virus (HIV) (2). With the widespread availability of antiretroviral therapy (ART), the aging of the population, and changes in the demographics of AIDS transmission, the life expectancy of people living with HIV (PLWH) has significantly improved, and AIDS in older age groups is becoming increasingly common (3, 4). Globally, by 2030, it is projected that the proportion of HIV-infected individuals aged 50 years or older will increase to 73% (5). Although the life expectancy of PLWH has increased, they often bear a higher burden of chronic diseases due to the effects of HIV infection and the side effects of antiretroviral therapy, such as cardiovascular disease (6) and cognitive impairment (7), presenting new challenges for the management of these conditions. Previous research has shown that physical activity is crucial for improving health outcomes in older adults living with HIV (8). Meanwhile, the WHO recommends that asymptomatic HIV-infected individuals or those with stable conditions on ART and a viral load below the detection limit for more than 6 months should complete at least 150 min of moderate-intensity aerobic exercise per week (9). However, many PLWH still do not follow current guideline recommendations: for example, globally, only 20 to 50% of PLWH meet the WHO exercise recommendations for HIV-infected individuals (10–12). Moreover, PLWH also show low levels of adherence in exercise interventions (13). Therefore, there is a need to explore more attractive and effective strategies to encourage PLWH to engage in physical activity.

PA is an established lifestyle intervention that plays a crucial role in promoting health (14). In HIV-infected individuals, a substantial body of research evidence has revealed the benefits of PA, including the effective prevention of cardiovascular disease development (15). Cross-sectional studies have also reported significant improvements in neurocognitive function and daily functioning among younger PLWH who regularly engage in physical activity (16, 17). The benefits of regular exercise extend beyond the serological and physiological changes often observed in the HIV population and also help address depressive symptoms and mental health issues in PLWH (18). Despite acknowledging the benefits of exercise, the physical activity levels of middle-aged and older adults living with HIV are not only lower than those of their HIV-negative counterparts but also more likely to fail to reach the WHO-recommended levels (10, 11). As the age of HIV-infected individuals increases, physical activity and exercise should be incorporated into their comprehensive management to improve overall function and quality of life in this older infected population. Given that participation and adherence to physical activity is a challenge for middle-aged and older PLWH, it is important to understand the facilitators and barriers to behavior change in this population.

To our knowledge, quantitative methods have been used in existing studies to identify factors associated with PA participation among PLWH. A review of 45 survey studies showed that the physical activity levels of HIV/AIDS patients are constrained by factors such as older age, lower education levels, and reduced CD4 cell counts, while high self-efficacy, motivation, and perceived benefits are considered important facilitators of PA (19). Similarly, many qualitative studies have also explored factors influencing physical activity among HIV-infected individuals, such as self-efficacy, social support levels, and accessibility of exercise environments (20–22). Although these qualitative studies have explored factors influencing exercise among PLWH, the methodologies of these studies vary, and the lack of systematic evaluation and synthesis makes it difficult to provide references for practice. Therefore, this study adopted the qualitative systematic review method of the Joanna Briggs Institute (JBI) to summarize qualitative studies on PA participation among middle-aged and older adults living with HIV, in order to understand the key factors that hinder exercise behavior and the motivation for sustained exercise in this population, with the expectation of better providing more effective, comprehensive, and personalized professional care from the perspective of those living with the infection.

### 1.1 Aim

The aim of this study is to critically evaluate and synthesize qualitative research on the attitudes, experiences, and facilitators and barriers to participation in physical activity among middle-aged and older adults living with HIV.

## 2 Methods

### 2.1 Protocol registration and reporting guidelines

This systematic review utilized the Enhancing Transparency in Reporting the Synthesis of Qualitative Research (ENTREQ) checklist (Supplementary File 1) to report the process and results of the synthesis (23). It was also registered on the International Prospective Register of Systematic Reviews (PROSPERO) with the registration number CRD42024498010.

### 2.2 Searching strategy

Two researchers developed the search strategy to identify reports on factors related to physical activity among middle-aged and older adults living with HIV. Four databases were searched: Web of Science, Embase, PubMed, and CINAHL, each with a tailored search strategy. The search terms used in the databases included: “HIV, ““Human immunodeficiency,” “Acquired immunodeficiency syndrome,” “Acquired immune deficiency syndrome,” “AIDS,” “older adult,” “elder,” “senior,” “aging,” “aged,” “older person,” “older people,” “Middle-aged,” “older adult(s),” “active,” “exercise,” “physical activity,” and “physical behavior.” Results were limited to journal articles or theses published in English before December 2024. Conference abstracts, research protocols, and social commentaries were manually excluded. Additionally, we hand-searched the reference lists of target articles to identify eligible studies. The search strategies used are detailed in Supplementary File 2.

### 2.3 Eligibility criteria

Studies were included if they met all the following criteria: (1) Utilized a qualitative research design (e.g., phenomenology, grounded theory, case study, ethnography, action research, or other qualitative methods); (2) Recruited HIV-infected individuals aged 45 years or older; (3) Investigated the barriers and facilitators to physical activity (PA) participation. In this review, barriers were defined as obstacles or negative feedback encountered by participants, as well as challenges they faced in engaging in or participating in PA. Facilitators were described as factors or positive feedback that attracted participants to engage in PA; (4) Were peer-reviewed articles or theses published in English.

Studies were excluded if they met any of the following criteria: (1) Used a mixed-methods design with inseparable qualitative data; (2) Full-text articles were unavailable; (3) Included middle-aged and older adults living with HIV who had physical disabilities, as their conditions might affect their levels of physical activity.

### 2.4 Study selection

Study selection followed the guidelines for systematic reviews (24). The initial search results from the databases were imported into Endnote 21 for screening. After removing duplicates, the titles and abstracts were reviewed to exclude reviews, quantitative studies, and articles unrelated to the topic. Finally, the most eligible studies were included after reading the full texts. At each screening stage, at least two trained reviewers (ZYL, ZXT, SD) independently assessed the articles. Any disagreements were resolved through discussion to reach a consensus.

### 2.5 Quality appraisal of eligible studies

Two independent researchers (ZYL and ZXT) rigorously evaluated the methodological quality of the included studies using the Checklist for Qualitative Research (Critical Appraisal tools for use in JBI Systematic Reviews) (25). The evaluation criteria consisted of 10 items, each with three possible judgments: “yes,” “no,” or “unclear.” ZYL and ZXT independently conducted the quality assessment, and any contentious studies were discussed with a third researcher (CF) to reach a consensus. Studies were categorized as low quality if the proportion of “yes” responses to the 10 evaluation criteria was below 60%; medium quality if the proportion was between 70 and 90%; and high quality if all items received a “yes” response (26).

### 2.6 Data extraction and synthesis

Two researchers (ZYL and ZXT) independently extracted data from the included studies using the JBI meta-aggregation approach (25). This method categorized the extracted results from each study based on thematic similarity and further synthesized these categories. The extracted data included authorship, country of publication, year of publication, participant characteristics (number, age), study objectives, primary research methods, and reported barriers and facilitators. ZYL and ZXT conducted the data synthesis analysis. The extracted facilitators and/or barriers were organized into descriptive main themes and sub-themes through an inductive approach.

## 3 Results

### 3.1 Searching results

**Figure 1:**
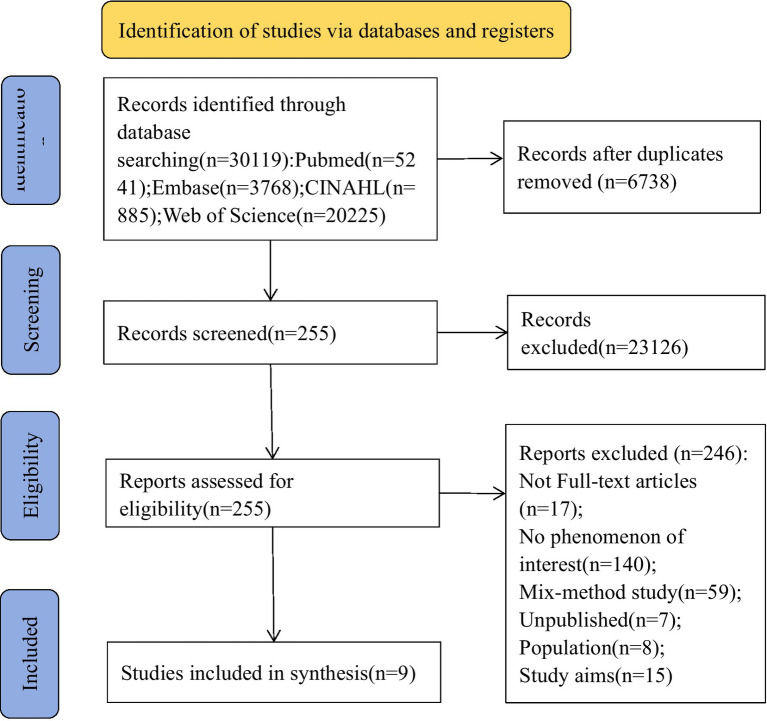
PRISMA flowchart for literature search.

As shown in Figure 1, the search yielded a total of 30,119 articles. After removing duplicates, 23,381 articles were screened by title and abstract, resulting in the exclusion of 23,126 articles. Finally, 255 full-text articles were reviewed, and 9 qualitative studies were included in this systematic review. The complete list of included studies is provided in Supplementary File 3.

### 3.2 Study characteristics

Table 1 provides a description of the included studies. Among the 9 qualitative studies included, 3 used focus group methods, while the remaining 6 employed semi-structured interviews. Half of the studies (n = 5) were conducted in Canada (27–31), 3 in the United States (32–34), and 1 in Africa (35). Across all 9 studies, a total of 148 middle-aged and older PLWH were included, of whom 37 were women, with the mean age ranging from 46 to 70 years (Table 2).

**Table 1:** Characteristics of studies included.

Study	Country	Aim	Characteristic of participants(sample size, age)	Methodological and sampling approach	Method of data collection and analysis
Simonik et al. (27)	Canada	To explore readiness to engage in exercise among people living with HIV and multimorbidity.	*N* = 14 age (y): mean = 50 (46–53) % female: 35.7	• Descriptive qualitative design; • Convenience sampling	• Face-to-face semi-structured interviews; • Thematic analysis.
Montgomery et al. (28)	Canada	To explore the experiences of engaging in a community-based exercise programme (CBEP) from the perspective of people living with HIV (PLWH).	*N* = 11 age (y): mean = 52(48–60) % female: 9	• Descriptive qualitative design; • Convenience sampling	• Semistructured interviews; • Thematic analysis.
Nguyen et al. (32)	United States	To sought feedback from potential end users when developing the exercise component of the intervention.	*N* = 27 age (y): mean = 54.4 (SD ± 4.8) % female: 19	• Qualitative design; • Convenience sampling	• Focus group sessions; • Thematic analysis.
Quigley et al. (29)	Canada	To use the Theoretical Domains Framework to investigate the barriers and facilitators to participation in exercise of older people living with HIV.	*N* = 12 age (y): mean = 56.6 (SD ± 8.8) % female: 25	• Qualitative design; • Convenience sampling	• In-depth, semi-structured interviews; • Thematic analysis.
Johs et al. (33)	United States	To examine the differences in perceived barriers and benefits of exercise among older PLWH by self-identified exercise status.	*N* = 29 age (y): above 50% female: 13.8	• Qualitative design; • Convenience sampling	• Focus group sessions; • Thematic analysis.
Neff et al. (34)	United States	To examined the barriers and facilitators to exercise among older PLWH initiating an exercise regimen.	*N* = 19 age (y): mean = 56.9 (SD ± 5.4) % female: 0	• Qualitative design; • Convenience sampling	• Focus group sessions; • An inductive and deductive analytic toolkit for applied behavioral analysis
Homayouni et al. (30)	Canada	To explore experiences participating in a group-based physiotherapist (PT)-led exercise programme among people living with HIV and complex multimorbidity.	*N* = 10 age (y): mean = 58(53–68) % female: 20	• Descriptive qualitative design; • Convenience sampling	• Semistructured interviews; • Qualitative descriptive analysis
Chetty et al. (35)	South Africa	To explore the perceptions of OPLWH about physical activity and exercise.	*N* = 16 age (y): above 50% female: 43.8	• Phenomenological, qualitative design; • Convenience sampling	• In-depth interviews; • Thematic analysis.
Sahel-Gozin et al. (31)	Canada	To explore experiences engaging in exercise from the perspectives of women living with HIV	*N* = 10 age (y): mean = 54(49–57) % female: 100	• Qualitative descriptive design; • Purposive and snowball sampling	• Online semi-structured interviews; • Descriptive thematic analysis

**Table 2:** Summary of the methodological quality and dependability of eligible studies.

Study ID	1	2	3	4	5	6	7	8	9	10	Overall	Total percent “yes” response	Dependability rating
Simonik et al. (27)	Y	Y	Y	Y	Y	N	N	Y	Y	Y	B	80%	Moderate
Montgomery et al. (28)	Y	Y	Y	Y	Y	Y	Y	Y	Y	Y	A	100%	High
Nguyen et al. (32)	Y	Y	Y	Y	Y	N	N	Y	Y	Y	B	80%	Moderate
Quigley et al. (29)	Y	Y	Y	Y	Y	N	N	Y	Y	Y	B	80%	Moderate
Johs et al. (33)	Y	Y	Y	Y	Y	N	N	Y	Y	Y	B	80%	Moderate
Neff et al. (34)	Y	Y	Y	Y	Y	N	N	Y	Y	Y	B	80%	Moderate
Homayouni et al. (30)	Y	Y	Y	Y	Y	Y	Y	Y	Y	Y	A	100%	High
Chetty et al. (35)	Y	Y	Y	Y	Y	Y	Y	Y	Y	Y	A	100%	High
Sahel-Gozin et al. (31)	Y	Y	Y	Y	Y	Y	N	Y	Y	Y	B	90%	Moderate

The item of Critical Appraisal tools for use in JBI Systematic Reviews: 1. Is there congruity between the stated philosophical perspective and the research method? 2. Is there congruity between the research methodology and the research question or objectives? 3. Is there congruity between the research methodology and the methods used to collect data? 4. Is there congruity between the research methodology and the representation and analysis of data? 5. Is there congruity between the research methodology and the interpretation of results? 6. Is there a statement locating the researcher culturally or theoretically? 7. Is the influence of the researcher on the research, and vice- versa, addressed? 8. Are participants, and their voices, adequately represented? 9. Is the research ethical according to current criteria or, for recent studies, and is there evidence of ethical approval by an appropriate body? 10. Do the conclusions drawn in the research report flow from the analysis, or interpretation, of the data? Note: “Y” means “Yes,” “N” means “No”.

### 3.3 Methodological quality and level of dependability

All studies scored highly on participant representation (item 8) and on the conclusion that the results reported in the studies were derived from data analysis or interpretation (item 10). All studies demonstrated good consistency between the research methods and the research objectives. Three of the included studies were rated as high quality, while the remaining six were rated as medium quality.

### 3.4 Meta-aggregation

**Figure 2:**
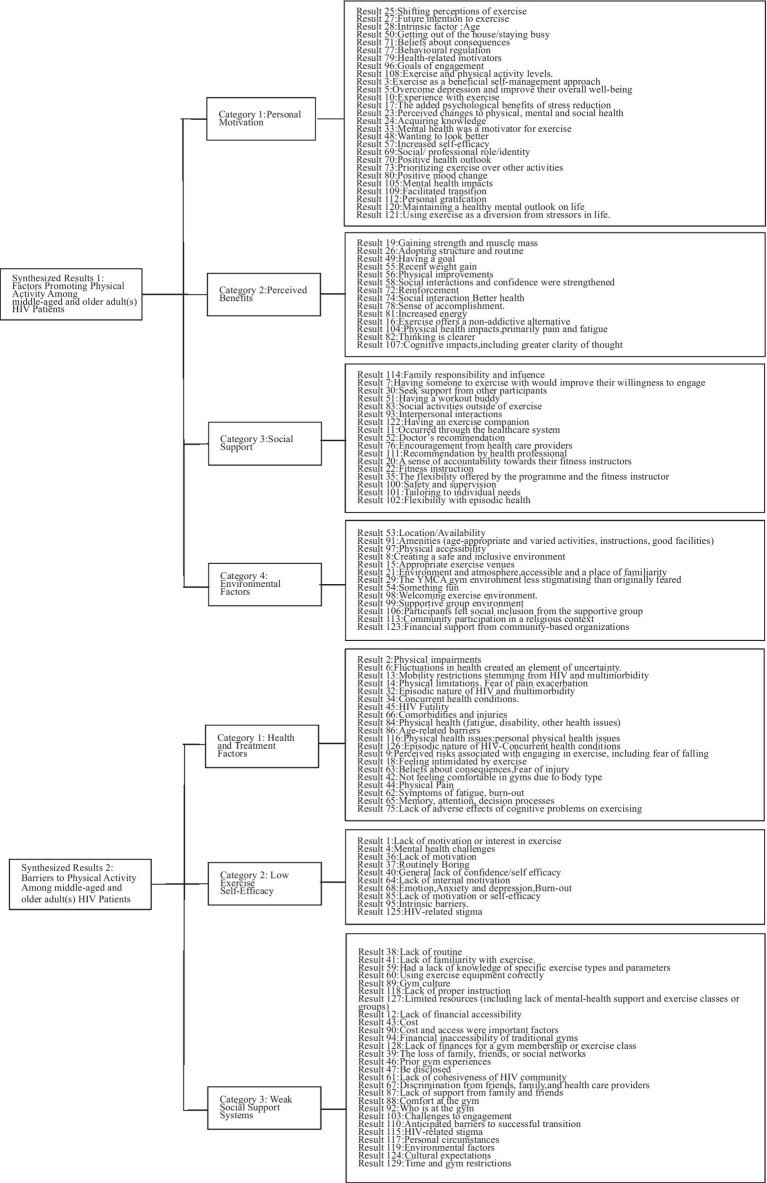
Synthesized results.

From the 9 studies included in the meta-aggregation, a total of 130 results were rated as “credible” or “clear.” The 130 study results were aggregated into 7 categories, which were then synthesized into 2 overarching results. The process of generating and summarizing the categories of facilitators and barriers to physical activity among middle-aged and older adults living with HIV is detailed in Supplementary File 4 (Figure 2).

#### 3.4.1 Synthesized results 1: factors promoting physical activity among older adult(s) HIV patients

Engagement in physical activities among older adult(s) HIV patients is significantly influenced by a mixture of personal motivation, perceived physical and mental benefits, strong social support systems, and favorable environmental conditions.

This synthesis illustrates how internal drives, perceived health improvements, external support, and environmental accessibility collectively encourage older adult(s) HIV patients to engage in and benefit from regular physical activity.

##### 3.4.1.1 Personal motivation

Central to initiating and maintaining an exercise regimen is the individual’s intrinsic motivation. Patients often cite a deep-seated belief in the health benefits of exercise as a primary motivator. This includes a commitment to combat the physical declines associated with aging and a desire to maintain mental wellness. Notable sentiments among patients include exercising to stay active and healthy as they age, and using physical activity as a strategy to prevent or mitigate depressive episodes (28–31).

##### 3.4.1.2 Perceived benefits

The tangible and immediate benefits of exercise, such as increased energy, reduced reliance on pain medication, and mental clarity, are key motivators for older adult(s) HIV patients. Reports from the patients underscore that physical activity contributes to a stronger and lighter feeling post-exercise, alongside clearer cognitive functions, which underscore the holistic benefits of regular physical engagement (30, 32, 33, 35).

##### 3.4.1.3 Social support

The role of a supportive social network, encompassing family members, peers, medical professionals, and fitness experts, cannot be overstated. Emotional and logistical support from family and encouragement from peers enhance the exercise experience. Professional guidance from healthcare providers and fitness instructors also significantly boosts patients’ confidence and commitment to regular physical activity. This comprehensive support system not only encourages initial participation but also sustains long-term engagement in physical activities (28, 30, 31, 35). However, social networks not only provide positive support but also exert social pressure. The study by Sahel-Gozin et al. (31) found that women living with HIV commonly worry about being identified as HIV-positive when considering participation in physical activity in public settings such as gyms. This fear of stigmatization leads them to avoid public exercise venues. Therefore, intervention strategies should focus on enhancing the positive role of social networks while mitigating the social pressure arising from stigma and discrimination.

##### 3.4.1.4 Environmental factors

Accessibility and the quality of the physical environment play crucial roles. The convenience of location and the welcoming nature of exercise venues, such as gyms, contribute to higher levels of activity. Facilities that are easy to access and provide a positive, supportive atmosphere are more likely to attract and retain older adult(s) HIV patients, fostering an ongoing commitment to physical health (30, 34).

#### 3.4.2 Synthesized results 2: barriers to physical activity among older adult(s) HIV patients

The participation of older adult(s) HIV patients in physical activities is hindered by several critical barriers including health and treatment-related issues, low exercise self-efficacy, and weak social support systems.

This synthesis provides a detailed exploration of the barriers faced by older adult(s) HIV patients regarding physical activity, grounding each point with direct references and expanding on the original text to provide a clearer, more comprehensive understanding of these impediments.

##### 3.4.2.1 Health and treatment factors

Physical limitations and complications associated with HIV and its treatment are primary barriers. Patients often discuss how co-morbidities such as chronic pain and diabetes restrict their mobility and activity levels. Fear of injury due to age-related physical fragility also contributes to reluctance in engaging in exercise. Additionally, side effects from medications, particularly fatigue, significantly deter patients from participating in regular physical activity (27, 32, 35).

##### 3.4.2.2 Low exercise self-efficacy

Many older adult(s) HIV patients express a lack of belief in their ability to engage in regular exercise, which is compounded by a general loss of interest or motivation as they age. This is often linked to long-standing negative emotions and a lack of personal conviction that they can maintain an exercise regimen, leading to sporadic or non-existent physical activity (27, 29, 33).

##### 3.4.2.3 Weak social support systems

Insufficient support from social networks and a lack of professional guidance on exercise are significant barriers. Many patients cite the absence of adequate instruction on exercise modalities and the skills required as obstacles. Financial constraints also play a role, with the cost of gym memberships or exercise programs often being prohibitive. Additionally, the lack of companionship and encouragement from peers further diminishes their motivation to engage in physical activities (29, 31, 35).

## 4 Discussion

This review explored the attitudes and experiences of middle-aged and older adults living with HIV toward exercise and physical activity, and identified the main barriers and facilitators. According to the results of the meta-aggregation, personal motivation is the initiating factor for promoting exercise among infected individuals. The perceived health benefits of exercise for both physical and mental well-being are key intrinsic factors that drive exercise behavior. High levels of social support are also an essential component in promoting participation in physical activity (PA) among this population. The higher the level of social support, the more conducive it is to the persistence of exercise behavior among middle-aged and older adults living with HIV. Additionally, environmental factors also influence the exercise motivation of infected individuals to some extent. Conversely, disease and treatment factors impose physical limitations on infected individuals, leading to concerns about exercise. Prolonged exposure to negative emotions such as depression results in a lack of confidence in exercise, leading to low exercise self-efficacy. Moreover, a weak social support system also contributes to poor adherence to exercise behaviors among infected individuals. Therefore, a comprehensive and multi-level assessment of the individual circumstances of middle-aged and older adults living with HIV is necessary. In future clinical research, this will facilitate better recommendations or guidance for middle-aged and older infected individuals to engage in any type of structured exercise or physical activity interventions.

The synthesis results emphasize that personal motivation among middle-aged and older adults living with HIV is an important factor in promoting PA. To attract this population to participate, it is necessary to consider their motivations and the significance of their involvement in PA. Studies have found that personal exercise motivation is associated with self-efficacy, which has long been considered one of the strongest correlates of exercise behavior among HIV-infected individuals. It is typically defined as a person’s belief in their ability and confidence to complete a task or a series of actions (19, 36). Individuals with low exercise self-efficacy have less awareness of the benefits of exercise and less confidence in their exercise capabilities, and thus, have less motivation to start exercising (37). In contrast, those with high self-efficacy are more likely to utilize self-care symptom management strategies after HIV infection, thereby generating health-protective behaviors (38). Ye et al.’s study confirmed that the occurrence of dyslipidemia among infected individuals is closely related to their low levels of physical activity and other unhealthy lifestyle habits (39). It is recommended that healthcare providers inform HIV-infected individuals of the importance of lifestyle changes during consultations, and enhance their exercise beliefs and confidence at an early stage. They should also clearly communicate that regular exercise is one of the important measures for managing cardiovascular disease risk factors among HIV-infected individuals (15).

A high perception of exercise benefits is a powerful facilitator for middle-aged and older adults living with HIV to engage in physical activity. The health achievements obtained through exercise serve as motivation for this population to persist in exercise, which is consistent with the conclusions of Oliveira et al. (40). The reason may be that when exercise brings about an overall improvement in health for middle-aged and older adults living with HIV, they become convinced that exercise can produce valuable health benefits, thereby gaining confidence to continue exercising. Pullen and Moore’s studies mentioned that reducing opioid dependence through exercise is an additional benefit that HIV-infected individuals gain from participating in exercise (41, 42). Therefore, healthcare providers should encourage HIV-infected individuals to express the health achievements they gain from exercise, provide positive feedback, and promote the development of regular exercise habits. It is worth noting that some middle-aged and older adults living with HIV report benefiting from the potential cognitive benefits of exercise (43). Related studies have also found that the effects of exercise on improving cognitive function and cardiometabolic status are more motivating for infected individuals to persist in exercise, thereby forming a virtuous cycle and achieving better health outcomes (44). It is recommended that healthcare providers regularly urge middle-aged and older adults living with HIV and provide timely personalized feedback strategies to help them reach a state of spontaneous and sustained exercise.

Social networks can provide positive support and companionship, yet they may also potentially carry stigmatizing social pressures. Nevertheless, family and peer support are among the most common facilitators for HIV-infected individuals (45). Most middle-aged and older patients with chronic diseases who engage in exercise require support and companionship (46). In Dos Santos’ systematic review, it was pointed out that exercise interventions primarily focused on family-based aerobic and/or resistance training for 12 to 48 weeks may effectively promote PA participation among HIV-infected individuals, thereby improving their cardiopulmonary health and physical strength (47). Similarly, other studies have shown that exercise programs involving peer-led or peer-supported initiatives can promote and maintain PA adherence among middle-aged and older adults not infected with HIV (48). Fortunately, professional guidance from healthcare providers and fitness instructors also significantly enhances the confidence of middle-aged and older adults living with HIV in engaging in regular physical activity. The flexibility of fitness guidance can motivate older adults to continue exercising (28, 30), which is similar to the findings of Netz’s study. On one hand, due to the decline in physical function among middle-aged and older adults and the significant increase in age-related fitness plans, a “one-size-fits-all” approach to exercise guidance may overlook individual differences. Accurate personalized assessments and exercise plans can formulate the best exercise prescriptions and encourage middle-aged and older adults to participate in PA (49). On the other hand, evidence suggests that a trustworthy provider-patient relationship helps health personnel monitor and promote the success of PA interventions (50, 51). Therefore, future interventions could draw on the Kanyakla model (52) to develop peer-led group exercise interventions that strengthen the positive functions of social networks, while simultaneously mitigating the social pressures associated with stigma.

The most common barriers are health and treatment factors related to HIV and its treatment. Physical limitations have already been identified as a common barrier to PA participation among HIV-infected individuals (53). Previous studies have shown that chronic pain resulting from multimorbidity is common among middle-aged and older adults living with HIV (54), and high pain perception and severity of pain have been identified as major barriers to PA participation among this population (55). Due to the combined effects of aging and HIV infection, fear of physical injury and pain leads to greater exercise apprehension among middle-aged and older adults living with HIV, which was also identified as another barrier in our synthesis (29, 33). Middle-aged and older adults hope that evidence-based exercise recommendations tailored to their age and physical condition, considering the unique needs of this population, can be developed (56, 57). Increasing the diversity of PA programs to account for individual factors, including comorbidities, is supported by the results of this review. Additionally, side effects of medications significantly hinder infected individuals from participating in regular physical activity. A cross-sectional study in the United States reported that the top three side effects of HIV treatment medications were fatigue (70.72%), diarrhea (62.96%), and insomnia (58.97%) (58). Fatigue leads to reduced motivation among infected individuals, consistent with conclusions from related studies indicating that health issues are often direct factors affecting an individual’s exercise persistence (59). In contrast, infected individuals in Gray et al.’s study perceived exercise-induced fatigue as “benign” and short-lived (60). Some studies have pointed out that 150 min per week of moderate-intensity walking can improve fatigue among HIV-infected individuals (61). Therefore, future research could focus on the relationship between moderate-intensity exercise (such as jogging, brisk walking, etc.) and fatigue experiences among infected individuals.

A weak social support system is also a potential barrier, such as cold weather and limited availability of time (28, 31). For many middle-aged and older adults living with HIV, adverse weather is a hidden barrier and a factor that prevents them from participating in outdoor activities. This result is supported by similar studies showing that extreme weather conditions can reduce the proportion of HIV patients traveling and visiting clinics (62). New research indicates that telemedicine-based remote exercise programs can provide accessible and effective methods to mitigate the impact of weather, allowing middle-aged and older adults living with HIV to engage in regular physical exercise at home (63). Another barrier to PA participation is financial constraints, especially gym memberships. Lack of funds often leads to middle-aged and older adults being unable to afford fitness expenses and giving up exercise. This finding is supported by Pascual’s study, which demonstrated the association between per capita income and lack of physical activity, and further adjusted the accessibility of sports facilities to promote exercise participation among middle-aged and older adults in the community (64). Additionally, the enthusiasm of middle-aged and older adults living with HIV for PA is closely related to the geographical location of fitness venues. Gyms that are conveniently located, especially those closer to home, are more likely to be preferred by this population (30, 34). A descriptive study pointed out that traffic hazards are the only environmental predictor of PA participation among urban-dwelling older adults living with HIV (65). Gyms that are closer to home may support PA by reducing traffic hazards and safety concerns among middle-aged and older patients. Moreover, a friendly and inclusive cultural atmosphere in gyms also encourages PA participation among infected individuals. Psychological perception is a factor that needs to be considered. By enhancing the perceived well-being of HIV-infected individuals in gyms, their exercise adherence can be improved (36).

The results of this review also reported low self-efficacy among middle-aged and older adults living with HIV in participating in PA programs (27, 29, 33). These studies found that this population often experiences long-term negative emotions such as depression, losing interest and motivation in exercise, and being unable to establish or maintain exercise beliefs. Due to the dual stigma of HIV and aging, middle-aged and older adults often experience social isolation related to stigma and loneliness, preventing them from fully integrating into social activities (66). Differences in perceived stigma and discrimination can be observed between male and female HIV-infected individuals. Female infected individuals, due to their physiological vulnerability, experience stronger negative self-image, verbal discrimination, and physical violence (67, 68), which may lead to lower participation rates among women compared to men. This conclusion is consistent with Vancampfort’s study, which found that higher levels of internalized stigma among people living with HIV are associated with lower levels of physical activity (69). Additionally, middle-aged and older adults living with HIV may lack time to participate in PA due to family responsibilities and caregiving obligations (31). However, Warren-Jeanpiere’s study mentioned that caregiving can serve as a motivation for middle-aged and older women living with HIV to maintain health and has a positive impact on their self-management capabilities (70). This may be due to cultural expectations and social roles that lead women to face more complexities in this area. Researchers should further investigate ways to enhance the self-efficacy of middle-aged and older women living with HIV to promote their exercise participation and improve overall health outcomes among this population.

Translating the barriers and facilitators identified through qualitative research into concrete intervention strategies represents a critical direction for future inquiry. In recent years, a growing number of intervention studies have begun to address physical activity among middle-aged and older adults living with HIV. In a related effort, Oursler er al (63) employed a telehealth intervention that directly circumvented barriers related to transportation, environmental safety, and stigma, thereby offering a feasible alternative for individuals who face challenges accessing public spaces. Burk et al. (71) integrated exercise, nutrition, and social components, directly responding to the issue of social isolation. Collectively, these intervention approaches further support the practical implementation of diverse exercise modalities and barrier-free environmental design. Clinicians should therefore offer multiple, adaptable physical activity options based on an individual’s physical condition, environmental context, and psychological concerns, rather than adhering to a single prescriptive model.

### 4.1 Limitations and prospects

Although this study ensured the rigor of the systematic review of qualitative research, there are still some limitatins. First, this review included only nine primary studies, reflecting the current scarcity of research on factors influencing physical activity among middle-aged and older adults living with HIV. This limitation undermines both the reliability and the generalizability of the present review. Moreover, the majority of studies included in this review were conducted in developed countries, with only one study from a developing country. Therefore, the findings may not be fully applicable to middle-aged and low-income countries. Additionally, in our review, middle-aged and older women living with HIV accounted for only 25% of the study population. Thus, the facilitators and barriers identified for this group should be interpreted with caution. Finally, although physical activity (PA) is increasingly recognized as important, this review focused specifically on the facilitators of and barriers to PA and did not systematically examine dietary behaviors. Existing evidence suggests that unhealthy dietary habits and low levels of PA often co-occur among people living with HIV, jointly affecting their metabolic health and cardiovascular risk (72). Therefore, future research should consider simultaneously exploring the cognitive, attitudinal, and behavioral barriers related to both physical activity and diet in order to develop a more comprehensive understanding of lifestyle management in this population.

## 5 Conclusion

In summary, the aim of this study was to better understand the barriers and facilitators to PA among middle-aged and older adults living with HIV from their perspectives. Moreover, this study identified barriers and facilitators at multiple levels, including individual, family, environmental, and societal factors, providing key insights for future researchers to improve PA in this population. However, it is important to note that similar studies from low- and middle-income countries are scarce. Therefore, future research should consider these aspects. We recommend that future studies investigate the sustainability and scalability of different intensities of PA and explore the specific impacts on various subgroups of middle-aged and older adults living with HIV to promote their physical, psychological, and cognitive health.
