# Supplementary material for: Facilitators and barriers to physical activity in middle-aged and older adult(s) HIV infected persons: a systematic review of qualitative studies
Source: Front Public Health. 2026 Jun 2;14:1809117. doi: 10.3389/fpubh.2026.1809117 (PMC13268978; doi:10.3389/fpubh.2026.1809117)
Supplement: Supplementary file 1 [file Supplementary_file_1.docx]

**Supplementary file 1 ENTREQ checklist (Enhancing transparency in reporting the synthesis of qualitative research)**

| **No. Item** | **Guide Questions/Description** | **Reported on Page** |
| --- | --- | --- |
| 1. Aim | The aim of this study is to critically evaluate and synthesize qualitative research on the attitudes, experiences, and facilitators and barriers to participation in physical activity among middle-aged and older adults living with HIV. | P4 |
| 2. Synthesis methodology | Identify the synthesis methodology or theoretical framework which underpins the synthesis, and describe the rationale for the choice of methodology (e.g. meta-ethnography, thematic synthesis, critical interpretive synthesis, grounded theory synthesis, realist synthesis, meta-aggregation, meta-study, framework synthesis) | P6 |
| 3. Approach to searching | The search was pre-planned. Comprehensive search strategies were undertaken to seek all available studies. | Supplementary file-2 |
| 4. Inclusion criteria | Included studies used widely accepted qualitative data collection methods, with well-described methodology, including for example interviews, focus groups, direct observation, and participatory action research. Included studies also needed to have provided a clear description of recognized qualitative data analysis methods (e.g., grounded theory, narrative analysis, content analysis, thematic analysis).  Studies were excluded if they met any of the following criteria: (1) Used a mixed-methods design with inseparable qualitative data; (2) Full-text articles were unavailable; (3) Included middle-aged and older adults living with HIV who had physical disabilities, as their conditions might affect their levels of physical activity. | P5 |
| 5. Data sources | Two researchers developed the search strategy to identify reports on factors related to physical activity among middle-aged and older adults living with HIV. Four databases were searched: Web of Science, Embase, PubMed, and CINAHL, each with a tailored search strategy. The search terms used in the databases included: "HIV," "Human immunodeficiency," "Acquired immunodeficiency syndrome," "Acquired immune deficiency syndrome," "AIDS," "older adult," "elder," "senior," "aging," "aged," "older person," "older people," "Middle-aged," "elderly," "active," "exercise," "physical activity," and "physical behavior." Results were limited to journal articles or theses published in English before December 2024. Conference abstracts, research protocols, and social commentaries were manually excluded. Additionally, we hand-searched the reference lists of target articles to identify eligible studies. The search strategies used are detailed in Supporting Information S2. | P4 |
| 6. Electronic Search strategy | Supplementary file-2 describes the literature search | Supplementary file-2 |
| 7. Study screening methods | Study selection followed the guidelines for systematic reviews. The initial search results from the databases were imported into Endnote 21 for screening. After removing duplicates, the titles and abstracts were reviewed to exclude reviews, quantitative studies, and articles unrelated to the topic. Finally, the most eligible studies were included after reading the full texts. At each screening stage, at least two trained reviewers (ZYL, ZXT, SD) independently assessed the articles. Any disagreements were resolved through discussion to reach a consensus. | P5 |
| 8. Study characteristics | Table 1 presents the characteristics of the included studies (author(s), year of publication, country, population, number of participants, data collection, methodology, and analysis ). | Table 1 |
| 9. Study selection results | A flow diagram using PRISMA guidelines for reporting of systematic reviews is presented in Figure 1 in reporting of the selection process and results. | Figure 1 |
| 10. Rationale for appraisal | A minimum of two trained reviewers （ZYL,ZXT,SD） independently evaluated the methodological rigor of the included literature following the Checklist for Qualitative Research (Critical Appraisal tools for use in JBI Systematic Reviews) (Lockwood et al., 2015). This checklist includes ten items, and each item is evaluated with “yes”, “no” or “unclear”. When the evaluation results conflicted, the third researcher (CF), with expertise in qualitative research, decided finally. A study was included if the item of it achieved a minimum of 60% “yes” to guarantee the study showed acceptable quality. Studies were considered to possess acceptable quality if 60% of items were answered “yes”, to possess good quality if 70-90% of items were answered “yes”, and to have high quality if 100% of items were answered “yes” (Talley et al., 2021). | P5 |
| 11. Appraisal items | 2020 version of Checklist for Qualitative Research (Critical Appraisal tools for use in JBI Systematic Reviews) | Table 2 |
| 1. Appraisal   process | The appraisal was conducted independently by two independent reviewers. The two reviewers discussed if consensus was required. When the evaluation results conflicted, the third researcher (CF) decided. | P5 |
| 13. Appraisal results | Appraisal results are presented in Table 3 | Table 2 |
| 14. Data extraction | This approach entails extracting findings from each study, then categorizing them through their similarity in meaning. Then, we subjected these categories to further synthesis to generate more comprehensive findings called synthesized findings. A finding is defined as a verbatim extract of the author’s analytical interpretation of the results or data. As a finding is extracted, the level of ‘plausibility’ should be allocated based on the reviewers’ assessment of the degree of fit, or congruency between the data and the accompanying exemplar quote. There are three levels of plausibility. A finding was rated as “unequivocal” if the congruence of the finding and the illustration accompanied was beyond a reasonable doubt; as “credible” if a clear association between them was lacking; as “unsupported” if the findings were not supported by the data. Only unequivocal and credible findings were included. Not supported findings were not presented in the synthesis or the results (Lockwood et al., 2015). | P6 |
| 15. Software | Results of database searches were first imported into the reference management software program Endnote 21. After the removal of duplicates, titles and abstracts were read for the assessment of eligibility. | P5 |
| 16. Number of reviewers | A minimum of two trained reviewers (ZYL, ZXT, SD, CF) | P5 |
| 17. Coding | JBI meta-aggregation did not use the technique of coding |  |
| 18. Study comparison | The process of aggregation involves the synthesis of findings by categorizing them through the similarity in meaning. Then, we subject these categories to a synthesis to generate more comprehensive findings. | P7 |
| 19. Derivation of themes | We subjected these categories to further synthesis to generate more comprehensive findings called synthesized findings. Only unequivocal and credible findings were included. Not supported findings were not presented in the synthesis or the results (Lockwood et al., 2015). | Figure 2 |
| 20. Quotations | “No. of finding file” provides findings and quotations from the primary studies to illustrate themes and constructs, and identify whether the quotations were participant quotations of the author’s interpretation. | Supplementary file- No. of finding |
| 21.Synthesis output | Synthesis output is presented in Supplemental file-4. | Supplementary file-4 Summary of study findings, categories, and synthesized categories to generate synthesized findings on the on the facilitators and barriers to physical activity among middle-aged and elderly HIV-infected individuals |

Reference: Tong A, Flemming K, McInnes E, Oliver SA, Craig J. Enhancing transparency in reporting the synthesis of qualitative research: ENTREQ. BMC Medical Research Methodology 2012, 12:181.

NA means 'not applicable'
