# Supplementary material for: Facilitators and barriers to physical activity in middle-aged and older adult(s) HIV infected persons: a systematic review of qualitative studies
Source: Front Public Health. 2026 Jun 2;14:1809117. doi: 10.3389/fpubh.2026.1809117 (PMC13268978; doi:10.3389/fpubh.2026.1809117)
Supplement: Supplementary file 2 [file Supplementary_file_2.docx]

## Supplementary file 2 Searching strategies

| **Pubmed** |  |  |
| --- | --- | --- |
| Search number | Query | Results |
| 1 | HIV[MeSH Terms] AND (2000:2024[pdat]) | 77,380 |
| 2 | AIDS[MeSH Terms] AND (2000:2024[pdat]) | 22,849 |
| 3 | ((HIV[Title/Abstract]) OR (AIDS[Title/Abstract]) OR (Acquired immunodeficiency syndrome[Title/Abstract]) OR (Acquired immune deficiency syndrome[Title/Abstract]) OR (Human immunodeficiency[Title/Abstract])) AND (2000:2024[pdat]) | 355,695 |
| 4 | #1 OR #2 OR #3 | 361,242 |
| 5 | aged[MeSH Terms] AND (2000:2024[pdat]) | 2,516,102 |
| 6 | elderly[MeSH Terms] AND (2000:2024[pdat]) | 2,516,102 |
| 7 | aging[MeSH Terms] AND (2000:2024[pdat]) | 192,226 |
| 8 | (older adult[Title/Abstract]) OR (elder[Title/Abstract]) OR (senior[Title/Abstract]) OR (aging[Title/Abstract]) OR (aged[Title/Abstract]) OR (older person[Title/Abstract]) OR (older people[Title/Abstract]) OR (Middle-aged[Title/Abstract]) OR (elderly[Title/Abstract]) | 1,112,129 |
| 9 | #5 OR #6 OR #7 OR #8 | 3,278,758 |
| 10 | physical activity[MeSH Terms] AND (2000:2024[pdat]) | 228,395 |
| 11 | ((active[Title/Abstract]) OR (exercise[Title/Abstract]) OR (physical activity[Title/Abstract]) OR (physical behavior[Title/Abstract])) AND (2000:2024[pdat]) | 1,302,347 |
| 12 | #10 OR #11 | 1,379,559 |
| 13 | #4 AND #9 AND #12 | 5,241 |

| **WOS** |  |  |
| --- | --- | --- |
| Search number | Query | Results |
| 1 | (TI=(HIV) OR TI=(AIDS) OR TI=(Acquired immunodeficiency syndrome) OR TI=(Acquired immune deficiency syndrome) OR TI=(Human immunodeficiency)) AND (DOP=(2000-01-01/2024-12-31)) | 572,413 |
| 2 | ((AB=(HIV) OR AB=(AIDS) OR AB=(Acquired immunodeficiency syndrome) OR AB=(Acquired immune deficiency syndrome) OR AB=(Human immunodeficiency))) AND (DOP=(2000-01-01/2024-12-31)) | 1,319,282 |
| 3 | #1 OR #2 | 1,508,159 |
| 4 | (TI=(older adult) OR TI=(elder) OR TI=(senior) OR TI=(aging) OR TI=(aged) OR TI=(older person) OR TI=(older people) OR TI=(Middle-aged) OR TI=(elderly))AND (DOP=(2000-01-01/2024-12-31)) | 1,449,216 |
| 5 | (AB=(older adult) OR AB=(elder) OR AB=(senior) OR AB=(aging) OR AB=(aged) OR AB=(older person) OR AB=(older people) OR AB=(Middle-aged) OR AB=(elderly))AND (DOP=(2000-01-01/2024-12-31)) | 7,144,754 |
| 6 | #4 OR #5 | 7,610,082 |
| 7 | (TI=(active) OR TI=(exercise) OR TI=(physical activity) OR TI=(physical behavior) )AND (DOP=(2000-01-01/2024-12-31)) | 1,188,355 |
| 8 | (AB=(active) OR AB=(exercise) OR AB=(physical activity) OR AB=(physical behavior) )AND (DOP=(2000-01-01/2024-12-31)) | 4,401,089 |
| 9 | #7 OR #8 | 4,738,187 |
| 10 | #3 AND #6 AND #9 | 20,225 |

| **Embase** |  |  |
| --- | --- | --- |
| Search number | Query | Results |
| 1 | (hiv:ab,ti OR aids:ab,ti OR 'acquired immunodeficiency syndrome':ab,ti OR 'acquired immune deficiency syndrome':ab,ti OR 'human immunodeficiency':ab,ti)AND [2000-2024]/py | 474,893 |
| 2 | ('older adult':ab,ti OR elder:ab,ti OR senior:ab,ti OR aging:ab,ti OR aged:ab,ti OR 'older person':ab,ti OR 'older people':ab,ti OR 'middle aged':ab,ti OR elderly:ab,ti) AND [2000-2024]/py | 1534939 |
| 3 | (active:ab,ti OR exercise:ab,ti OR 'physical activity':ab,ti OR 'physical behavior':ab,ti) AND [2000-2024]/py | 1729075 |
| 4 | #1 AND #2 AND #3 | 3768 |

| **CINAHL** |  |  |
| --- | --- | --- |
| Search number | Query | Results |
| 1 | AB(HIV OR AIDS OR "Acquired immunodeficiency syndrome" OR "Acquired immune deficiency syndrome" OR "Acquired immune deficiency syndrome" OR "Human immunodeficiency") | 85,419 |
| 2 | AB("older adult" OR elder OR senior OR aging OR aged OR "older person OR "older people" OR "Middle-aged" OR "elderly" ) | 302,265 |
| 3 | AB(active OR exercise OR "physical activity" OR "physical behavior") | 270,292 |
| 4 | #1 AND #2 AND #3 | 885 |
