# Supplementary material for: Facilitators and barriers to physical activity in middle-aged and older adult(s) HIV infected persons: a systematic review of qualitative studies
Source: Front Public Health. 2026 Jun 2;14:1809117. doi: 10.3389/fpubh.2026.1809117 (PMC13268978; doi:10.3389/fpubh.2026.1809117)
Supplement: Supplementary file 3 [file Supplementary_file_3.docx]

**Supplementary file 3 Full included studies**

1.Simonik A, Vader K, Ellis D, Kesbian D, Leung P, Jachyra P, et al. Are you ready? Exploring readiness to engage in exercise among people living with HIV and multimorbidity in Toronto, Canada: a qualitative study. BMJ Open. 2016;6(3):e010029.

2.Montgomery CA, Henning KJ, Kantarzhi SR, Kideckel TB, Yang CF, O'Brien KK. Experiences participating in a community-based exercise programme from the perspective of people living with HIV: a qualitative study. BMJ Open. 2017;7(4):e015861.

3.Quigley A, Baxter L, Keeler L, MacKay-Lyons M. Using the Theoretical Domains Framework to identify barriers and facilitators to exercise among older adults living with HIV. AIDS Care. 2019;31(2):163-8.

4.Homayouni TS, Ruth A, Abbott-Tate Z, Burger H, Rahim S, Murray C, et al. Experiences engaging in a group-based physiotherapist-led exercise programme for adults living with HIV and complex multimorbidity: a qualitative study. BMJ Open. 2021;11(7):e045158.

5.Sahel-Gozin N, Loutfy M, O'Brien KK. Exploring experiences engaging in exercise from the perspectives of women living with HIV: A qualitative study. PLoS One. 2023;18(6):e0286542.

6.Nguyen AL, Lake JE, Reid MC, Glasner S, Jenkins J, Candelario J, et al. Attitudes towards exercise among substance using older adults living with HIV and chronic pain. AIDS Care. 2017;29(9):1149-52.

7.Johs NA, Kellar-Guenther Y, Jankowski CM, Neff H, Erlandson KM. A qualitative focus group study of perceived barriers and benefits to exercise by self-described exercise status among older adults living with HIV. BMJ Open. 2019;9(3):e026294.

8.Neff HA, Kellar-Guenther Y, Jankowski CM, Worthington C, McCandless SA, Jones J, et al. Turning disability into ability: barriers and facilitators to initiating and maintaining exercise among older men living with HIV. AIDS Care. 2019;31(2):260-4.

9.Chetty L, Cobbing S, Chetty V. The perceptions of older people living with hiv/aids towards physical activity and exercise. AIDS Res Ther. 2022;19(1):67.
