# Supplementary material for: Facilitators and barriers to physical activity in middle-aged and older adult(s) HIV infected persons: a systematic review of qualitative studies
Source: Front Public Health. 2026 Jun 2;14:1809117. doi: 10.3389/fpubh.2026.1809117 (PMC13268978; doi:10.3389/fpubh.2026.1809117)
Supplement: Supplementary file 4 [file Supplementary_file_4.docx]

**Supplementary file 4 Summary of study finding, categories, and synthesized categories to generate synthesized findings on the facilitators and barriers to physical activity among middle-aged and elderly HIV-infected individuals.**

| Synthesized Results 1: Factors Promoting Physical Activity Among middle-aged and Elderly HIV Patients | | |
| --- | --- | --- |
| Findings [Credibility Rating] | Categories | Synthesized Category |
| 25.Shifting perceptions of exercise | combat the physical declines associated with aging | Personal Motivation |
| 27.Future intention to exercise |  |  |
| 28.Intrinsic factor :Age |  |  |
| 50.Getting out of the house/staying busy |  |  |
| 71.Beliefs about consequences |  |  |
| 77.Behavioural regulation |  |  |
| 79.Health-related motivators |  |  |
| 96.Goals of engagement |  |  |
| 108.Exercise and physical activity levels |  |  |
| 3.Exercise as a beneficial self-management approach | desire to maintain mental wellness |  |
| 5.Overcome depression and improve their overall well-being |  |  |
| 10.Experience with exercise |  |  |
| 17.The added psychological benefits of stress reduction |  |  |
| 23.Perceived changes to physical, mental and social health |  |  |
| 24.Acquiring knowledge |  |  |
| 33.Mental health was a motivator for exercise |  |  |
| 48.Wanting To Look Better |  |  |
| 57.Increased self-efficacy |  |  |
| 69.Social/ professional role/  identity |  |  |
| 70.Positive health outlook |  |  |
| 73.Prioritizing exercise over other activities |  |  |
| 80.Positive mood change |  |  |
| 105.Mental health impacts |  |  |
| 109.Facilitated transition |  |  |
| 112.Personal gratifcation |  |  |
| 120.Maintaining a healthy mental outlook on life |  |  |
| 121.Using exercise as a diversion from stressors in life. |  |  |
| 19.Gaining strength and muscle mass | increased energy | Perceived Benefits |
| 26.Adopting structure and routine |  |  |
| 49.Having a goal |  |  |
| 55.Recent weight gain |  |  |
| 56.Physical improvements |  |  |
| 58.Social interactions and confidence were strengthened |  |  |
| 72.Reinforcement |  |  |
| 74.Social interaction  .Better health |  |  |
| 78.Sense of accomplishment |  |  |
| 81.Increased energy |  |  |
| 16.Exercise offers a non-addictive alternative | reduced reliance on pain medication |  |
| 104.Physical health impacts,primarily pain and fatigue |  |  |
| 82.Thinking is clearer | mental clarity |  |
| 107.Cognitive impacts,including greater clarity of thought and improved concentration |  |  |
| 114.Family responsibility and infuence | encompassing family members | Social Support |
| 7.Having someone to exercise with would improve their willingness to engage | Encompassing peers |  |
| 30.Seek support from other participants |  |  |
| 51.Having a workout buddy |  |  |
| 83.Social activities outside of exercise |  |  |
| 93.Interpersonal interactions |  |  |
| 122.Having an exercise companion |  |  |
| 11.Initial exposure to exercise occurred  through the healthcare system during periods of health crises | medical professionals |  |
| 52.Doctor’s recommendation |  |  |
| 76.Encouragement from health care providers |  |  |
| 111.Recommendation by health professional |  |  |
| 20.A sense of accountability towards their fitness instructors and to themselves | fitness experts |  |
| 22.Fitness instruction |  |  |
| 35.The flexibility offered by the programme and the  fitness instructor |  |  |
| 100.Safety and supervision |  |  |
| 101.Tailoring to individual needs |  |  |
| 102.Flexibility with episodic health |  |  |
| 53.Location/Availability | The convenience of location | Environmental Factors |
| 91.Amenities (age-appropriate  and varied activities, instructions, good facilities) |  |  |
| 97.Physical accessibility |  |  |
| 8.Creating a safe and inclusive environment | the welcoming nature of exercise venues |  |
| 15.Appropriate exercise venues |  |  |
| 21.Environment and atmosphere,accessible and a place of familiarity |  |  |
| 29.The YMCA gym environment less stigmatising than originally feared |  |  |
| 54.Something fun |  |  |
| 98.Welcoming exercise environment |  |  |
| 99.Supportive group environment |  |  |
| 106.Participants felt social inclusion from the supportive group |  |  |
| 113.Community participation in a religious context |  |  |
| 123.Financial support from community-based organizations |  |  |
| Synthesized Results 2: Barriers to Physical Activity Among middle-aged and Elderly HIV Patients | | |
| Findings [Credibility Rating] | Categories | Synthesized Category |
| 2.Physical impairments | Physical limitations and complications | Health and Treatment Factors |
| 6.Fluctuations in health created an element of uncertainty |  |  |
| 13.Mobility restrictions stemming from HIV and multimorbidity |  |  |
| 14.Physical limitations. Fear of pain exacerbation |  |  |
| 32.Episodic nature of HIV and multimorbidity |  |  |
| 34.Concurrent health conditions |  |  |
| 45.HIV Futility |  |  |
| 66.Comorbidities and injuries |  |  |
| 84.Physical health (fatigue, disability, other health issues) |  |  |
| 86.Age-related barriers |  |  |
| 116.Physical health issues:personal physical health issues |  |  |
| 126.Episodic nature of HIV-Concurrent health conditions |  |  |
| 9.Perceived risks associated with engaging in exercise, including fear of falling | Fear of injury |  |
| 18.Feeling intimidated by exercise |  |  |
| 63.Beliefs about consequences,Fear of injury |  |  |
| 42.Not feeling comfortable in gyms due to body type | side effects from medications |  |
| 44.Physical Pain |  |  |
| 62.Symptoms of fatigue, burn-out |  |  |
| 65.Memory, attention,  decision processes |  |  |
| 75.Lack of adverse effects of cognitive problems  on exercising |  |  |
| 1.Lack of motivation or interest in exercise | lack of belief in their ability to engage in regular exercise | Low Exercise Self-Efficacy |
| 4.Mental health challenges |  |  |
| 36.Lack of motivation |  |  |
| 37.Routinely Boring |  |  |
| 40.General lack of confidence/self efficacy |  |  |
| 64.Lack of internal motivation |  |  |
| 68.Emotion,Anxiety and depression,Burn-out |  |  |
| 85.Lack of motivation or self-efficacy |  |  |
| 95.Intrinsic barriers |  |  |
| 125.HIV-related stigma |  |  |
| 38.Lack of routine | the absence of adequate instruction on exercise | Weak Social Support Systems |
| 41.Lack of familiarity with exercise |  |  |
| 59.Had a lack of knowledge of specific exercise types and parameters |  |  |
| 60.Using exercise equipment correctly |  |  |
| 89.Gym culture |  |  |
| 118.Lack of proper instruction |  |  |
| 127.Limited resources (including lack of mental-health support and exercise classes or groups) |  |  |
| 12.Lack of financial accessibility | Financial constraints |  |
| 43.Cost |  |  |
| 90.Cost and access were important factors |  |  |
| 94.Financial inaccessibility of traditional gyms |  |  |
| 128.Lack of finances for a gym membership or exercise class |  |  |
| 39.The loss of family, friends, or social networks | the lack of companionship and encouragement |  |
| 46.Prior gym experiences |  |  |
| 31.Weather and holidays |  |  |
| 47.Be disclosed |  |  |
| 61.Lack of cohesiveness of HIV community |  |  |
| 67.Discrimination from friends, family, LGBTQ community members, and health care providers |  |  |
| 87.Social factors (caregiver burden, lack of social group, lack of support from family  and friends) |  |  |
| 88.Comfort at the gym |  |  |
| 92.Who is at the gym |  |  |
| 103.Challenges to engagement |  |  |
| 110.Anticipated barriers to successful transition |  |  |
| 115.HIV-related stigma |  |  |
| 117.Personal circumstances |  |  |
| 119.Environmental factors |  |  |
| 124.Cultural expectations |  |  |
| 129.Time and gym restrictions |  |  |
| 130.Cold winter weather conditions |  |  |
